# Structure of human telomere G-quadruplex in the presence of a model drug along the thermal unfolding pathway

**DOI:** 10.1093/nar/gky1092

**Published:** 2018-11-08

**Authors:** Federico Bianchi, Lucia Comez, Ralf Biehl, Francesco D’Amico, Alessandro Gessini, Marialucia Longo, Claudio Masciovecchio, Caterina Petrillo, Aurel Radulescu, Barbara Rossi, Francesco Sacchetti, Federico Sebastiani, Nicolò Violini, Alessandro Paciaroni

**Affiliations:** 1 Dipartimento di Fisica e Geologia, Università di Perugia, 06123 Perugia, Italy; 2 IOM-CNR c/o Dipartimento di Fisica e Geologia, Università di Perugia, 06123 Perugia, Italy; 3 JCNS & ICS, Forschungszentrum Jülich GmbH, Leo-Brandt Strasse, 52425 Jülich, Germany; 4 Elettra-Sincrotrone Trieste, Strada Statale 14 - km 163,5 in AREA Science Park, 34149 Basovizza, Trieste, Italy; 5 JCNS at Heinz Maier-Leibnitz Zentrum (MLZ), Forschungszentrum Juelich GmbH, Lichtenbergstrasse 1, 85748 Garching, Germany; 6 Lehrstuhl für Physikalische Chemie 2, Ruhr-Universität Bochum, 44780 Bochum, Germany; 7 JCNS, Forschungszentrum Jülich GmbH, Leo-Brandt Strasse, 52425 Jülich, Germany

## Abstract

A multi-technique approach, combining circular dichroism spectroscopy, ultraviolet resonance Raman spectroscopy and small angle scattering techniques, has been deployed to elucidate how the structural features of the human telomeric G-quadruplex d[A(GGGTTA)3GGG] (Tel22) change upon thermal unfolding. The system is studied both in the free form and when it is bound to Actinomycin D (ActD), an anticancer ligand with remarkable conformational flexibility. We find that at room temperature binding of Tel22 with ActD involves end-stacking upon the terminal G-tetrad. Structural evidence for drug-driven dimerization of a significant fraction of the G-quadruplexes is provided. When the temperature is raised, both free and bound Tel22 undergo melting through a multi-state process. We show that in the intermediate states of Tel22 the conformational equilibrium is shifted toward the (3+1) hybrid-type, while a parallel structure is promoted in the complex. The unfolded state of the free Tel22 is consistent with a self-avoiding random-coil conformation, whereas the high-temperature state of the complex is observed to assume a quite compact form. Such an unprecedented high-temperature arrangement is caused by the persistent interaction between Tel22 and ActD, which stabilizes compact conformations even in the presence of large thermal structural fluctuations.

## INTRODUCTION

Guanine-rich DNA and RNA sequences are prone to fold into stable helical four-stranded structures called G-quadruplexes (1). These folds are the focus of a number of studies in both fundamental and applied research, from cancer biology and novel therapeutics (2) through to nanotechnology (3). Indeed, G-quadruplex motifs have been proposed to inhibit the reverse-transcriptase enzyme telomerase (4), which is up-regulated in over 85% of cancers but not in somatic cells (5). In addition, stable putative G-quadruplex forming sequences have been identified *in vivo* mainly within cancer genes in human chromatin (6). These findings greatly boosted the attention toward G-quadruplexes as attractive therapeutic targets for drugs like small molecule ligands that can stabilize their structure (1). On the structural point of view, G-quadruplexes consist of a stack of two or more G-tetrads, which are cation-coordinated squared planar arrangements made of four Hoogsteen hydrogen bonded guanine basis (1,2,7,8). G-quadruplexes display quite similar structural properties in terms of rise and twist of the right-handed helical motif, while any of the four G-tracts can have a parallel or anti-parallel direction (1). At the same time, also the geometry of the loops connecting the guanine segments can change, with these nucleotide strands assuming diagonal, lateral, or chain reversal conformations (9). The actual folded topology of G-quadruplexes strongly depends on a variety of factors, such as the oligonucleotide sequence, the particular cation used, the presence of crowding agents and the DNA concentration (10). Different folds can be separated by relatively small energy barriers, so that also switching between distinct conformers quite easily occurs (11). In fact, even rather small temperature variations may give rise to conformational changes and populate intermediate states in the path of G-quadruplexes toward unfolding (12). Finally, and certainly important for therapeutic applications, different conformers can be visited during the binding of G-quadruplexes with ligands (13). The basis belonging to TTA sequence must play a major role in conformational changes occurring upon complexation, due to their flexibility to form chain reversals, diagonal and lateral loops (14), and create effective platforms scaffolds for binding. The situation is even more intricate if one considers the conformational flexibility of the ligand, which can be an important factor to improve the stabilization of the complexes (15), and the fact that the ligand itself may drive dimerization of quadruplexes (16). The natural antibiotic and anticancer drug Actinomycin D (ActD) is a prototype of conformationally flexible ligands for nucleic acids, since it can finely adapt its structure to make stable complexes with extremely different systems, such as ds-DNA (17), DNA hairpin (18), oncogenic C-Myc promoter G-quadruplexes (19) and the human telomere AG_3_(TTAG_3_)_3_ (Tel22) quadruplex (20). In this context, the Tel22+ActD complex is a model system whose topological and large-scale structural properties deserve to be characterized in detail, as they arise from the combined conformational flexibilities of both the Tel22 and ActD components. The knowledge of the conformational landscape accessible to the Tel22+ActD complex can give hints on the way the interaction between G-quadruplex and ligand is related to and modulate the recognition and regulation processes.

Here, we deploy an integrated strategy based on complementary biophysical techniques to investigate the Tel22 quadruplex and the Te22+ActD complex. Circular dichroism (CD) spectroscopy, Ultraviolet Resonance Raman (UVRR) scattering and small angle X-ray and neutron scattering (SAXS and SANS) provide an accurate view of both the topological and the large-scale structural properties of Tel22 and Tel22+ActD in the path toward thermal melting.

We find direct structural evidence for ligand-promoted Tel22 dimerization, with ActD bound to the quadruplex by end-stacking upon the extremal G-tetrad. We show that the denaturation of Tel22 is a multistate process, with the topology of the intermediate free states affected by the presence of the drug. After melting the bound Tel22 is characterized by a quite compact structure and a residual stacking. This evidence for the existence of a high-temperature partially folded state for the complexed Tel22, analogous to the molten globule state of proteins, appears like a remarkable trait of the stabilizing action of ActD on human telomeric G-quadruplex.

## MATERIALS AND METHODS

### Sample preparation

The oligonucleotide sequence AG_3_(TTAG_3_)_3_ (Tel22) was purchased from Eurogentec (Belgium) and used without further purification. The lyophilized powder was dissolved in a 50 mM phosphate buffer at pH 7, 0.3 mM EDTA and 150 mM KCl. This solution was heated to 95°C for 5 min and then slowly cooled down to room temperature in ∼4 h. After this procedure, the samples were left at room temperature overnight. DNA concentration was determined from UV absorption measurements at 260 nm, using a molar extinction coefficient of 228 500 M^−1^ cm^−1^ (data provided by Eurogentec). Samples with two different concentrations were prepared: 45 μM for CD and UVRR measurements, 150 μM for SAXS studies and 450 μM for SANS experiments. Complexed samples were obtained by adding ActD from Sigma-Aldrich previously dissolved in the same phosphate buffer. Drug concentration was estimated as for DNA, using a molar extinction coefficient of 24500 M^−1^ cm^−1^ at 440 nm (21). Then, small moieties of the drug solution were added to the quadruplex solutions to reach the stoichiometric ratio of Tel22:ActD 1:2, for the 45 μM, the 150 μM and the 450 μM samples. The complexed samples were left overnight at room temperature to reach full complexation.

### Circular dichroism

Circular dichroism experiments were performed using Jasco J810 spectropolarimeter on the Tel22 and the Tel22+ActD samples. A 1 mm path-length quartz cuvette was used, in order to obtain the optimum signal-to-noise ratio. Spectra were collected by varying the temperature through a thermal bath from 30 to 82°C, with steps of 2°C. Each spectrum was collected in the range from 220 to 325 nm, with a scan speed of 50 nm/min.

### UVRR scattering

UVRR measurements were carried out at the IUVS beamline at Elettra Sincrotrone Trieste by exploiting a properly optimized synchrotron-based experimental setup (22). All of the samples were placed into a 10 mm path quartz cuvette for UVRR measurements. The spectra were excited at 250 nm and collected in a backscattered geometry by using a triple stage spectrometer with a spectral resolution of about ∼20 cm^−1^. Beam power measured on the samples was about 4 μW. For each sample, UVRR spectra were recorded in the temperature range from 30°C to 90°C, with steps of 4°C. Standard calibration measurements, such as those of buffer and a 45 μM drug solution, were performed in order to ensure that their contribution to the UVRR signal is negligible. To compare the Tel22 and Tel22+ActD spectra at a given temperature, the intensity of the OH stretching band of water at about 3400 cm^−1^ was used as a standard for the normalization of the experimental profiles.

### Small angle neutron scattering

SANS measurements of the samples were performed at the small-angle diffractometer KWS-2 of the Jülich Centre for Neutron Science at Heinz Maier-Leibnitz Center (FRM II reactor in Garching, Germany). Using an incident wavelength λ = 2.9 Å and suitable sample to detector distances, we explored a wavevector Q window from 0.02 to 1 Å^−1^. Measurements were acquired at 30°C for samples placed in quartz cells of 1 mm path length. Raw data were corrected for the instrument background, detector sensitivity, and scattering from empty cell, and finally calibrated on the absolute scale (cm^−1^) using a Plexiglas secondary standard. Both Tel22 and Tel22+ActD samples were measured for about 24 h to collect high quality data. It is worth of notice that samples were prepared without resorting to deuterated buffer, as for DNA the contrast provided by H_2_O is better than that of D_2_O. The strong incoherent background from hydrogenated buffer has been carefully subtracted.

### Small angle X-ray scattering

SAXS patterns were collected using an Anton Paar SAXSpace kratky camera at Forschungzentrum Juelich. In order to follow the thermal melting, both Tel22 and Tel22+ActD samples were measured from 40 to 90°C in steps of 10°C. A relatively low sample concentration of 150 μM was chosen to probe the same G-quadruplex topology as in CD measurements, while granting for a proper signal to noise ratio (23,24). To obtain good statistics and correctly subtract the solvent contribution, data were accumulated for two hours. We obtained the pair distance distribution function *p*(*r*) and the radius of gyration (*R*_g_) performing a calculation on the experimental scattering curves by using the GNOM software (25). *Ab initio* models were obtained, for the two samples at 40 and 90°C, by sequential use of the DAMMIF (26,27), DAMAVER (28) and DAMMIN (26) programs. Twenty models per entity were generated by a simulated annealing procedure with DAMMIF and consequently overlapped and averaged by the DAMAVER suite. Finally, refined shapes were obtained by fitting the averaged models to proper PDB models, using DAMMIN.

### Singular value decomposition

CD and UVRR spectra collected for the two samples were separately arranged into data matrices **A_i,j_**_._ Spectroscopic variables (wavelength for CD, Raman shift for UVRR) run through the *i* rows, while temperature runs through the *j* columns of the matrices. The *i*th row represents a melting profile at λ_i_, the *j*th column represents a measured spectrum at a fixed temperature *T*_*j*_. The four resulting matrices **A_i,j_**_._ were analyzed by singular value decomposition using the software Octave 4.0, obtaining the **U, S** and **V** matrices per each data set. The vectors of the **U** matrix are a linear combination of the spectral species that compose the measured spectra, **S** contains the singular values (i.e. the weights of each spectral component) and the vectors of the **V** matrix are a linear combination of the abundancies of the spectral species as a function of temperature. Criteria to determine the correct number of spectral species follow from the procedure by Gray and Chaires (29). The global fitting procedure was carried out with the software Gnuplot, by means of an ad-hoc script.

## RESULTS

### Circular dichroism reveals the topology changes of Tel22 and Tel22+ActD upon melting

**Figure 1.**
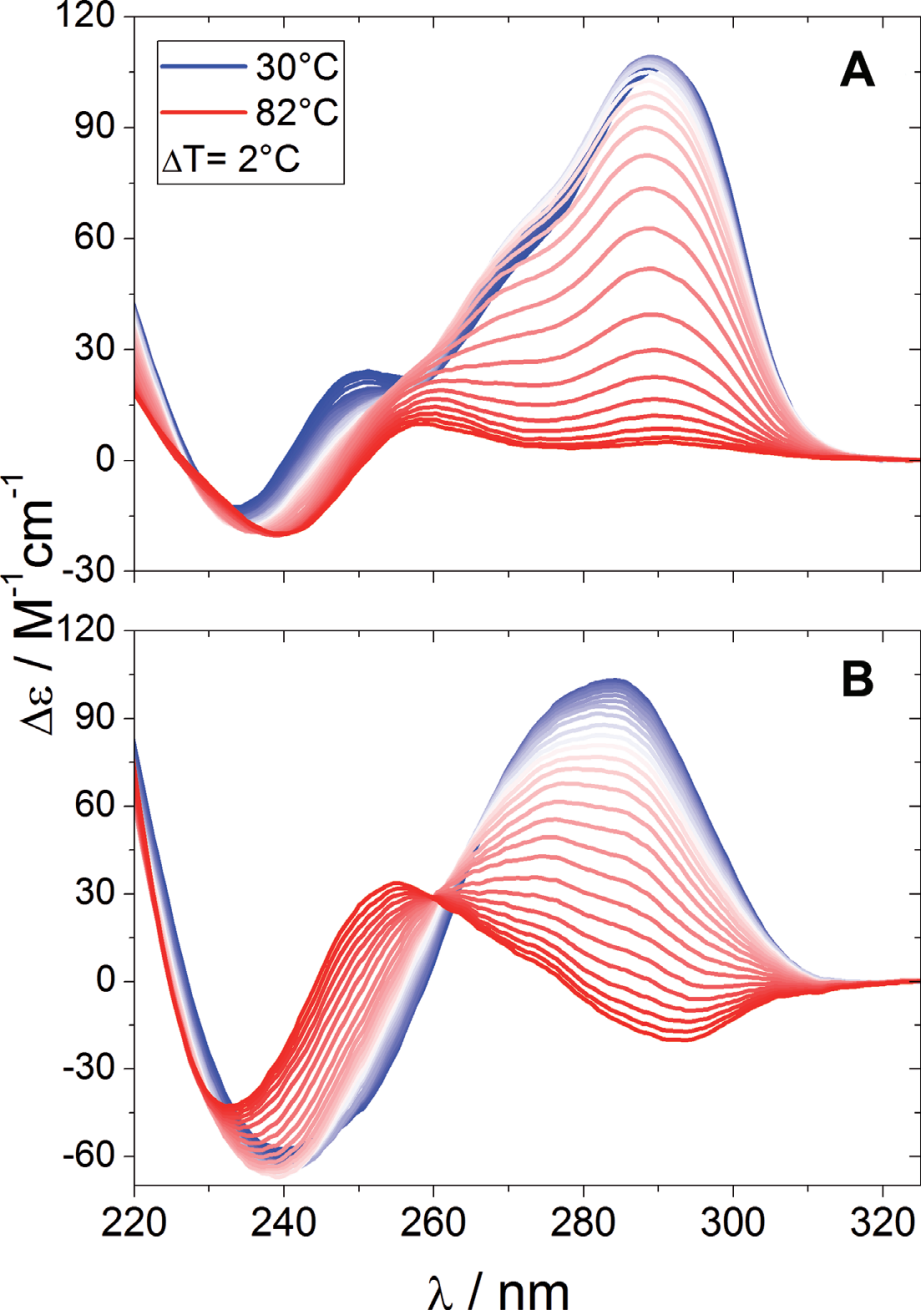
Temperature dependent unfolding assessed by CD. (**A**) Tel22 45μM in 150 mM KCl solution. (**B**) Tel22+ActD 1:2, 45 μM in 150 mM KCl solution. The ellipticity is normalized to strand concentration (22mer) and to path-length. In panel (**B**), the CD signal of Actinomycin D has been subtracted.

By using circular dichroism (CD), we probe the changes of topology for Tel22 and Tel22+ActD as a function of temperature on approaching the melting, as different geometry of quartet stacking gives rise to distinct spectroscopic signatures (9). Figures 1A and B show the spectra of Tel22 and Tel22+ActD respectively, in the temperature range 30°-82°C, with a step of Δ*T* = 2°C. At room temperature, the Tel22 spectrum is characterized by a maximum at ∼290 nm, a shoulder at ∼270 nm, and a minimum at ∼233 nm, such features being consistent with G-quadruplexes of hybrid type (9,30).

**Figure 2.**
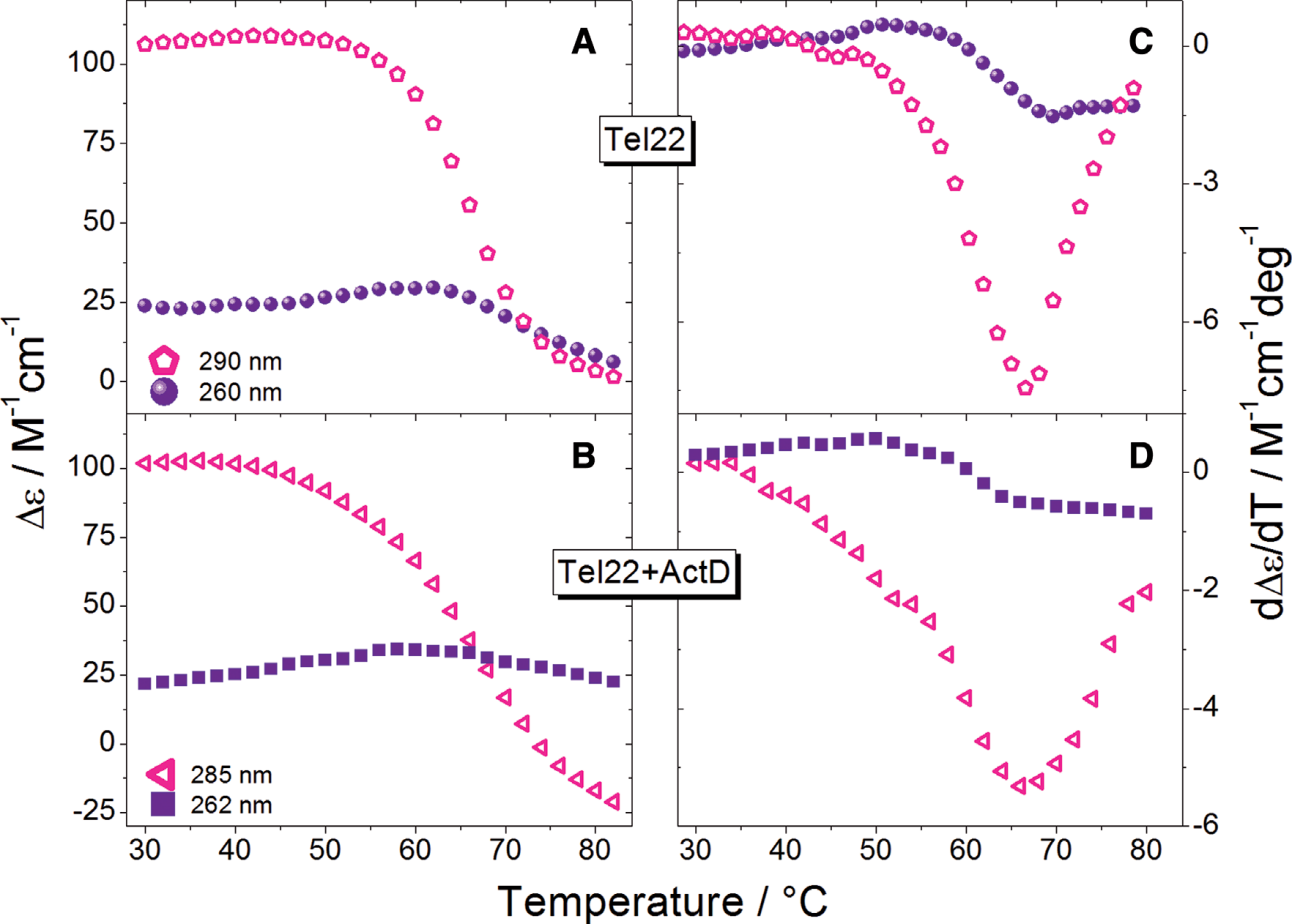
Panels (**A**) and (**B**): Single wavelengths melting profiles of Tel22 and Tel22+ActD extracted from the experimental data set, at two selected wavelengths. The CD curves display temperature dependence. Panels (**C**) and (**D**): First derivative of melting profiles curves in (A) and (B). The number of inflection points suggests the number of transitions along thermal melting process.

The presence of possible intermediate states can be enlightened by comparing the ellipticities measured at two different wavelengths, as in a simple two-state process their temperature dependence should be the same (12,31,32). Actually, Figure 2 shows that for both Tel22 and Tel22+ActD the ellipticities display distinct inflection points, which is a strong indication that the native state passes through intermediate states before attaining the denatured conformation.

To get quantitative information on the number of intermediate states and the thermodynamic parameters describing each step of the unfolding process, we performed the SVD analysis on the CD data. We found that four species are needed to describe the trend of the CD spectra over the whole temperature range for Tel22. In more detail, to accept a significant spectral species a cutoff value of 0.8 for the autocorrelation coefficients of the **V** matrix was chosen, which corresponds to a signal/noise ratio of 1. We found out that the four species with largest singular values satisfy this acceptance criterion and contribute to 98.56% of the total variance for the Tel22 (See Supplementary Table S1).

We verified that also in the present case, as proposed by Gray *et al.* (12), a sequential model with two intermediate states is more suited than one with two coexisting intermediate states, as it is congruent with the hypothesis that at least two conformers in tentatively equal amounts populate the structural heterogeneous distribution of Tel22 in KCl. Then we applied a model consisting of two sequential intermediate states, i.e. N ↔ I1 ↔ I2 ↔ U, where N, I1, I2 and U stand for native, first intermediate, second intermediate and unfolded states respectively (12). Notice that we considered here Tel22 with different concentration and buffer conditions compared to the case of ref (12).

At room temperature, the CD signal of Tel22+ActD displays a clear shift in the maximum from 290 to 285 nm as well as a change of the positive shoulder of Tel22 toward a minimum of ellipticity at 245 nm (see Supplementary Figure S1a). This trend has been explained as if the complexation of ActD induced a change in the quadruplex structure toward a parallel conformation (20). Quite interestingly, the high-temperature CD signal from Tel22-ActD is not featureless as in the case of Tel22, while showing a minimum at ∼250 nm and a maximum at 280 nm (see Supplementary Figure S1b). This trend is similar to the one of d(GpGp) dinucleotides (33), which indicates that even after unfolding there is residual stacking between guanine basis.

For Tel22+ActD we used the same two-intermediate sequential model as for free Tel22, because, also in this case, the native conformation has been suggested to consist of two structural isomers (20). We found that the four species with largest singular values contribute 98.04% of the total variance for the Tel22+ActD (see Supplementary Table S1). The excellent fit of the two-intermediate sequential model to the V vectors is displayed in Supplementary Figure S2 for Tel22 and in Supplementary Figure S3 for Tel22+ActD. The thermodynamic parameters obtained from the fit are reported in Table 1. Both the enthalpy changes and the melting temperatures of Tel22+ActD are slightly larger than Tel22, for all the transition steps found with the SVD analysis, thus confirming the stabilizing action of the drug.

**Table 1.** Thermodynamics parameters for the thermal melting of Tel22 and Tel22+ActD, obtained from SVD analysis on CD data of Figure 1

	G-quadruplex	Complex
Δ*H*_1_	−27.3 ± 1.5	−26.8 ± 1.4
*T*_m1_	38.5 ± 0.3	40.1 ± 0.3
Δ*H*_2_	−41.0 ± 2.5	−45.7 ± 3.0
*T*_m2_	60.3 ± 0.3	61.5 ± 0.3
Δ*H*_3_	−63.2 ± 3.8	−77.6 ± 5.4
*T*_m3_	68.9 ± 0.2	69.9 ± 0.2

Δ*H* is here expressed in kilocalories per mole, and *T* in degrees Celsius.

### UVRR spectroscopy reveals the molecular details of stacking of Tel22 and Tel22+ActD upon melting

**Figure 3.**
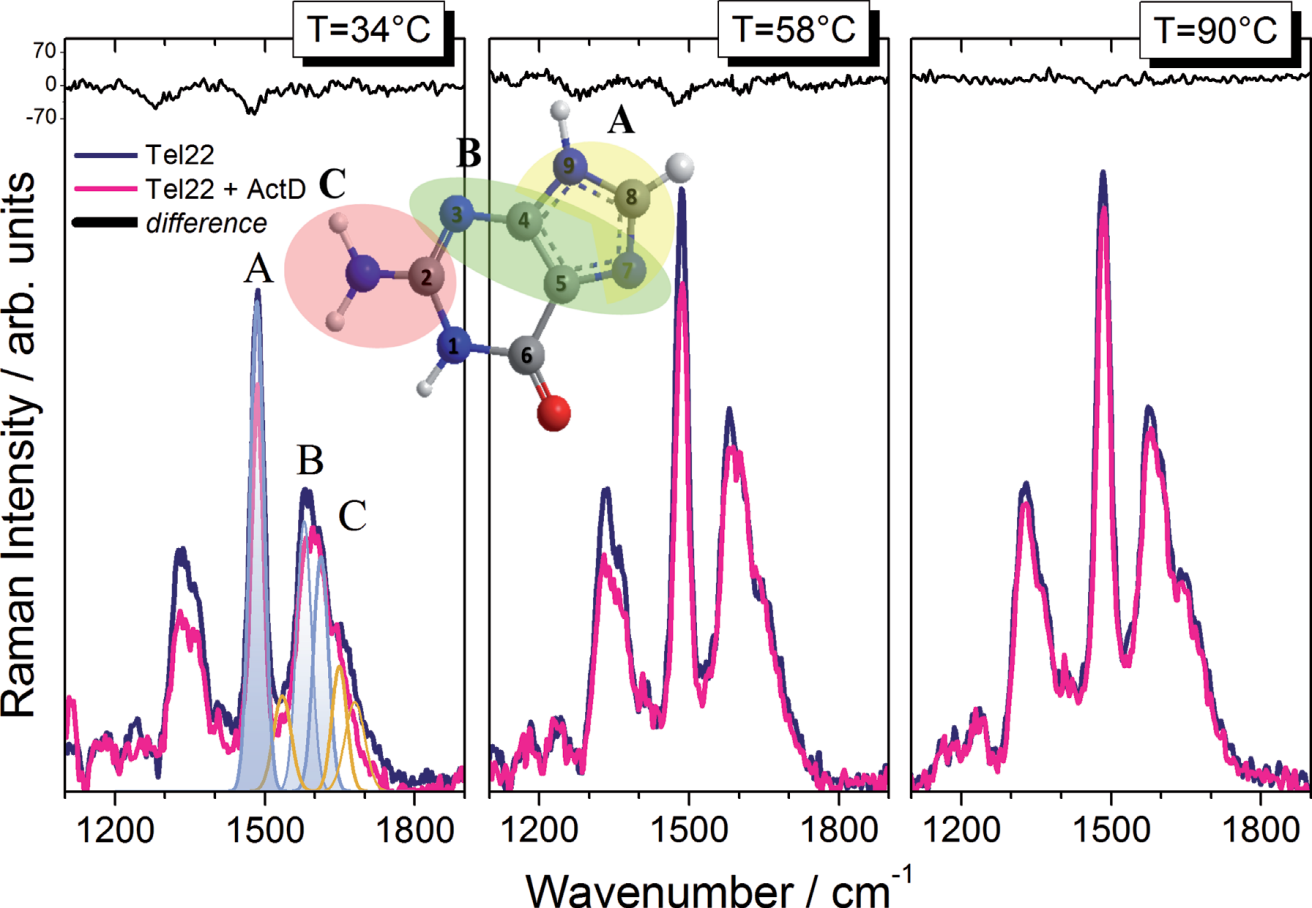
UVRR spectra of Tel22 and Tel22+ActD at three representative temperatures. In the left panel, an example of fit of the three main bands is reported: bands A, B and C are coloured in light blue; other underlying peaks are depicted by golden lines. The three bands (A = 1482 cm^−1^; B = 1578 cm^−1^, C = 1611 cm^−1^) are mainly attributable from normal in-plane modes of dG residues, as sketched in the central cartoon.

UVRR spectroscopy can selectively enhance the Raman intensities of the bands assigned to specific chromophores belonging to the system and has been used extensively to study concentration-dependent conformational transitions of nucleic acids (34) and complexation of quadruplexes with small molecules (35). In more detail, at the excitation wavelength λ_e_ = 250 nm used here, the contribution from in-plane vibrations of base residues is dominant in the UVRR spectra (36). The spectra measured for the Tel22 and the Tel22+ActD samples are shown in Figure 3 in the wavenumber range from 1000 to 1800 cm^−1^, and for three selected temperatures (the whole thermal cycles can be displayed in Supplementary Figure S4 for both systems). At the present excitation wavelength, the bands at ≈1482, 1578 and 1611 cm^−1^ (labelled as A, B and C respectively) are mainly attributable to vibrations of dG residues, with minor contributions from dA (36,37).

Since base stacking interactions, which give rise to absorption hypochromism, are known also to lead to the suppression of resonance Raman signal (38), the intensity of these bands gives specific information on the unstacking of G-tetrads. Interestingly, the bands A and B are less intense in the spectrum of Tel22+ActD than for Tel22, as evident also by inspection of the spectra difference shown at the top of the panels in Figure 3. This hypochromic effect suggests a more compact structure of the complex. Moreover, this trend confirms that ActD binding involves end-stacking upon the terminal G-tetrad of the quadruplex structures (20), as intercalation would cause the opposite behaviour of the bands intensity, i.e. hyperchromic effect. Since the hypochromicity persists also after unfolding, we argue that the base-stacking for Tel22+ActD complex is quite effective even when the system is progressively destabilized. In order to extract quantitative information of the temperature-behaviour of the spectral parameters of the bands A, B and C, a fitting of the experimental Raman profiles has been performed by using a minimum number of Gaussian functions, as shown in Figure 3, left panel, for Tel22.

**Figure 4.**
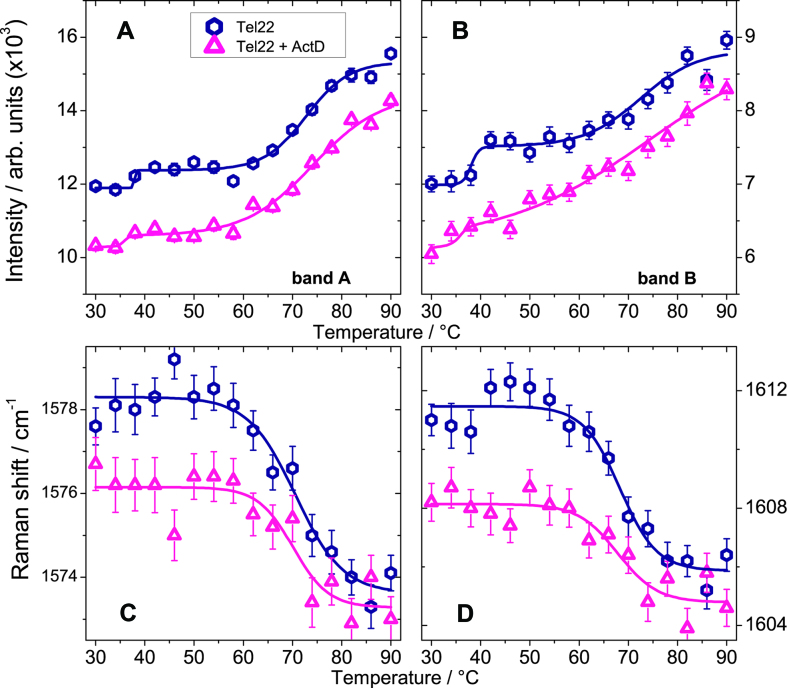
Top: Trend of the intensities of bands A, panel (**A**), and B, panel (**B**), as a function of temperature. Lines are only a guide for the eyes. Bottom: Temperature evolution of wavenumber position for the Raman bands B and C upon thermal melting. Panel (**C**): Band B for Tel22 (hexagones) and Tel22+ActD (triangles). Panel (**D**): Band C for Tel22 (hexagones) and Tel22+ActD (triangles). Lines are a guide for the eye.

The intensity of the band A (*I*_A_), which arises from the bending of C8–H, and the stretching of N9–C8 and C8–N7 (36,37) is reported as a function of the temperature in Figure 4A for Tel22 and Tel22-ActD. For both systems, a minor increase at about 35°C is followed by a much steeper rise between 60 and 80°C. The former rise is likely related to the N ↔ I1 transition seen by CD experiments, while the latter takes place just in correspondence to the third conformational change I2 ↔ U, more directly ascribed to the main unfolding of the G-quadruplex at about *T*_m_ = 70°C. No feature related to the transition I1↔I2 is observed, suggesting that it could mainly involve loop rearrangements that give small contributions to the Raman intensity of band A.

Since from the trend of *I*_A_ the presence of an intermediate state can be traced back, we apply also to UVRR data the SVD analysis. We considered a cutoff value of 0.7 for the autocorrelation coefficients of the **V** matrix to determine the number of significant spectral species, also taking into account for quality of the fit of the data. It turns out that the unfolding process involves one intermediate state, i.e. N ↔ I1 ↔ U (see Supplementary Table S2 for the corresponding singular values and autocorrelations coefficients). In comparing the results of the SVD analysis performed on data from UVRR with the one done on data from CD measurements, it has to be kept in mind that the latter technique is mainly sensitive to the G-quadruplex topology, while the former reports on local structural properties as reflected by the trend of the nucleobasis vibrational modes. This implies that the enthalpy changes estimated by SVD analysis from UVRR and CD data are not directly comparable. As for the conformational transition temperatures, the values from UVRR data compare well with *T*_m1_ and *T*_m3_ obtained by the SVD analysis of the CD spectra (see Table 2), suggesting that the topological changes occurring at those temperatures are also related to local rearrangements of base residues. Conversely, no local rearrangements of base residues is revealed in correspondence of the transition revealed by CD at *T*_m2_.

**Table 2.** Thermodynamics parameters for the thermal melting of Tel22 and Tel22+ActD, obtained from SVD analysis on UVRR data

	G-quadruplex	Complex
Δ*H*_1_	−34.5 ± 3.6	−46.7 ± 3.6
*T*_m1_	44.5 ± 0.3	37.6 ± 0.3
Δ*H*_2_	−72.6 ± 3.2	−40.9 ± 3.2
*T*_m2_	71.4 ± 0.5	73.1 ± 0.5

Δ*H* is here expressed in kilocalories per mole, and *T* in degrees Celsius.

Figure 4B shows the temperature evolution of the intensity of band B that is assigned to the stretching modes of bonds C4–N3, C5–C4 and N7–C5 in dG residue (36,37). The trend is similar to that already discussed for band A. In this case, larger statistical fluctuations are visible due to quite delicate fitting procedure involving the overlapping of band C. The intensity of this latter band C (not shown) increases linearly, probably due to the superposition of different premelting and melting transitions (39,40). The band B, whose position is sensitive to hydrogen bonding at exocyclic dG-N2H donor site of guanine, is located at 1578 cm^−1^, consistent with the formation of G-tetrads at room temperature (41). Its value clearly downshifts to ∼1574 cm^−1^ upon unfolding of the quadruplex, due to the substitution of inter-guanine with guanine-water hydrogen bonds (see Figure 4C). In the case of the complex Tel22+ActD, band B is centered at a slightly lower wavenumber of ∼1576 cm^−1^ at room temperature, probably because the inter-guanine Hoogsteen pairing is perturbed by the interaction with ActD. Actually, L-threonine residues of the ActD penta-peptide chains could be hydrogen bonded through their carbonyl oxygen atom and N–H group to the 2-aminogroup of guanine residue and N3 ring nitrogen respectively, as it happens in deoxyguanosine-ActD complex (42). Figure 4D reports the temperature-dependence of wavenumber position for band C, which is mainly attributable to the NH2 scissoring mode (36) of dG residues. The observed downshift from 1611 to 1606 cm^−1^ upon unfolding of Tel22 supports the sensitivity of band C to formation of G-quadruplex structures. Analogously to the case of band B, also the mode C is found at 1608 cm^−1^ in the spectrum of the complex at room temperature, a lower wavenumber than Tel22.

### SANS discloses the molecularity of the Tel22+ActD complex

**Figure 5.**
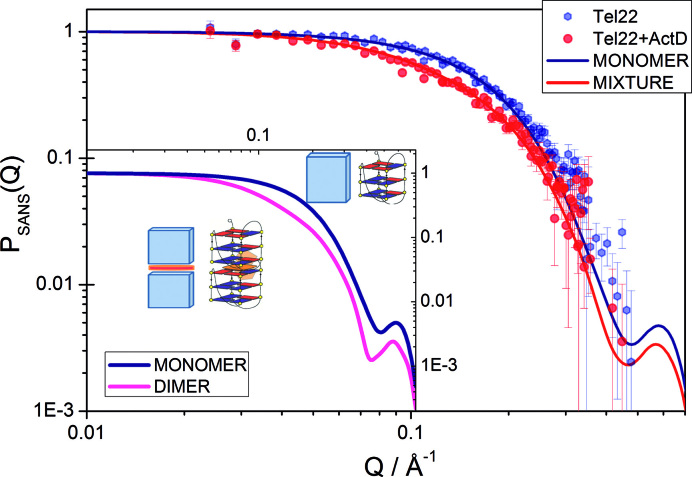
Form factors obtained from SANS measurements, for Tel22 (closed blue hexagones) and Tel22+ActD (closed red circles). The form factors of a parallelepiped representing the Tel22 monomer (blue line) and a mixture of parallelepiped monomers and dimers fitted to the complex data (red line) are also reported. Inset: Form factors of parallelepiped monomer (blue line) and parallelepiped dimer (magenta line), in comparison. For further details see S.I.

SANS data give precious information on the large scale structural features of Tel22 and Tel22+ActD complex. In Figure 5, it is seen that the measured form factor P_SANS_(Q) of Tel22 is excellently represented by the form factor of a squared parallelepiped with side 15 ± 1 Å and height 27 ± 1 Å. These data indicate that the G-quadruplexes are in a monomeric form in the present experimental conditions. On the other hand, to describe the quite different P_SANS_(Q) from Tel22+ActD we used a form factor consisting of a mixture of monomers (squared parallelepiped) and dimers (two adjacent squared parallelepipeds), representing Tel22+ActD complexes with molecularity 1:1 and 2:1 respectively. The drug-driven formation of dimers for the Tel22+ActD system has been suggested in the past on the basis of ITC measurements (20). The fitting procedure provides monomer characteristic sizes that are very similar to those of unbound Tel22 (15 ± 2 Å and height 28 ± 2 Å), suggesting a rather small contribution from the ligand to the complex molecular volume. In addition, we find that in the presence of ActD a fraction of 0.5 ± 0.1 G-quadruplexes undergoes dimerization. In Supplementary Figure S5 we show that also the low-Q absolute values of the SANS macroscopic cross section estimated within the hypothesis of a pure monomeric G-quadruplex form for the Tel22 sample and a mixture of monomers and dimers for the Tel22+ActD sample, are in very good agreement with the measured data. The radius of gyration calculated within the Guinier approximation (43), is 10.6±0.4 Å and 12.2±0.3 Å for respectively Tel22 and Tel22+ActD. The molecular volumes, which were estimated by exploiting the Porod invariant (43), turn out to be *V*_Tel22_ = (6.8 ± 0.3) × 10^3^ Å^3^ and *V*_Tel22+ActD_ = (10.1±0.4) × 10^3^ Å^3^. The value of *V*_Tel22_ is in quite good accord with the estimate of 6400 Å^3^ one can derive from the knowledge of the molecular weight and the partial specific volume usually assumed for G-quadruplexes of 0.55 ml/g (44). For more details about the modeling of SANS data, see the SI.

### SAXS shows the large-scale structural features of Tel22 and Tel22+ActD upon melting

**Figure 6.**
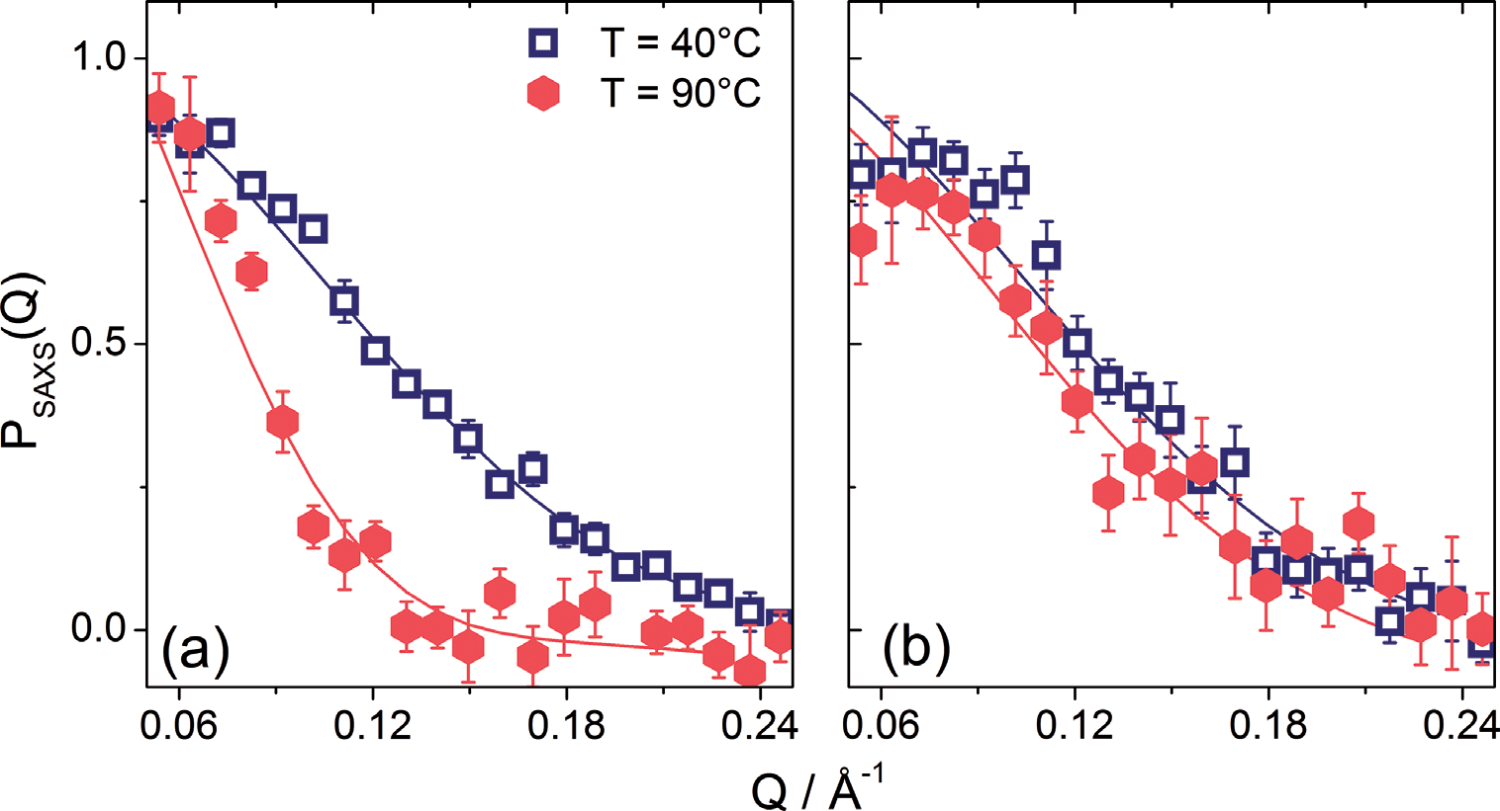
Form factors obtained from SAXS measurements at the indicated temperatures. Panel (**A**): P_SAXS_(Q) for Tel22. Panel (**B**): P_SAXS_(Q) for Tel22+ActD. Lines represent the fit done with dummy atom model (DAMMIN).

SAXS has been shown to be a powerful tool to study G-quadruplexes and G-tetrads aggregates in solution (45). Here, we have exploited SAXS, in a complementary way to SANS, to investigate the temperature trend of the Tel22 and Tel22+ActD large scale structural features. The measured form factors P_SAXS_(Q), for Tel22 and Tel22+ActD, at 40°C and 90°C, i.e. for the native and the unfolded states respectively, are reported in Figure 6. The decrease of P^U^_SAXS,Tel22_(Q) with increasing Q, which is much faster than P^N^_SAXS,Tel22_(Q), indicates that the characteristic size of the free G-quadruplex is definitely larger in its unfolded state than in the native one (46). On the other hand, the size of the complex at room temperature is almost the same as the one at high temperature, as indicated by the similar trend of P^U^_SAXS,Complex_(Q) and P^N^_SAXS,Complex_(Q).

**Figure 7.**
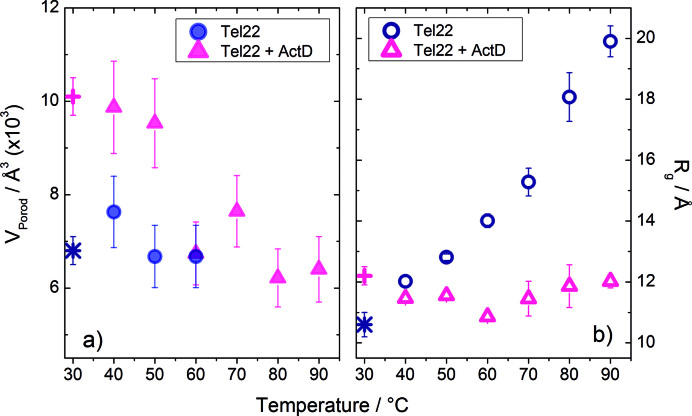
(**A**) Particle volume of Tel22 (solid circles) and Tel22+ActD (solid triangles), estimated by SAXS data as a function of the temperature through Porod invariant method. (**B**) Radius of gyration for Tel22 (open circles) and Tel22+ActD (open triangles) calculated by *p(r)* of Supplementary Figure S6. Values obtained by SANS measurements at 30°C (see text) are also reported for comparison (Tel22, star; Tel22+ActD, cross).

Further structural information can be obtained by calculating, from the form factor, the pair distance distribution function, *p(r)*, (Supplementary Figure S6). It provides a histogram of distances between all possible pairs of atoms within the free or the complexed Tel22 (see also Materials and Methods). As it can be seen, up to 60°C the *p*(*r*) of Tel22 shows a bell-shaped trend typical of globular compact biomolecules, while at higher temperatures an extended tail is more and more visible, which indicates the progressive unfolding of the quadruplex (46). On the contrary, the complex displays bell-shaped *p(r)* curves up to 70°C and only small tails at 80°C and 90°C, thus suggesting that quite compact structures are formed in the whole temperature range, even at temperatures above the major structural unfolding measured by CD and UVRR techniques. The Kratky plot confirms this point, as shown in Supplementary Figure S7. The shape of both the form factor and the pair distribution function from SAXS data does not allow one to obtain information on the molecularity of the investigated systems (see note in SI), this is why we resorted to the so-called Porod invariant to estimate the particle volume (see SI for details) (43). In the temperature range between 40°C and 60°C the molecular volume of Tel22 does not change and attains a value of (7.0 ± 0.7) × 10^3^ Å^3^, as shown in the panel (A) of Figure 7, while for higher temperature the volume cannot be calculated due to the failure of the Porod approximation. This estimated value is in agreement with the one from SANS measurement. On the other hand, the molecular volume of the complex is (9.8 ± 1.0) × 10^3^ Å^3^ up to 50°C, this trend being consistent with the presence of a fraction of ∼0.5 of Tel22 dimers in the monomer/dimer mixture, as also indicated by SANS data. The significant drop of the molecular volume just above 50°C to (7.0 ± 0.1) × 10^3^ Å^3^ testifies the separation of Tel22 dimers. Further quantitative and synthetic description of the compactness of the investigated systems is given by the radius of gyration, *R*_g_, which has been estimated from the calculated *p*(*r*). As shown in Figure 7B, at 40°C the radius of gyration of Tel22 and Tel22+ActD are rather in agreement with the values assessed by SANS. The *R*_g_ of the free quadruplex progressively increases with increasing the temperature, with a larger rate above 60°C. Quite remarkably, the value *R*_g_ = 20 ± 1 Å for Tel22 at 90°C is consistent with the SAXS experimental estimate for poly-T oligonucleotides of similar lengths (47). In this latter case the radius of gyration has been described in terms of the self-avoided random-walk (SAW) chain model: *R*_g_^SAW^ = *A*_0_*N*^ν^, with *A*_0_ = 3 Å, *N* is the number of nucleotides and *ν =* 0.62. Conversely, Tel22+ActD shows a radius of gyration of about 12 Å, which is quite constant in the whole investigated temperature range and very similar to that of the native Tel22 system. This result is congruent with the trend of the form factor and the pair distance distribution function and suggests that the destabilized state of the complex may have a partially folded compact structure even at the highest temperatures.

## DISCUSSION

**Figure 8.**
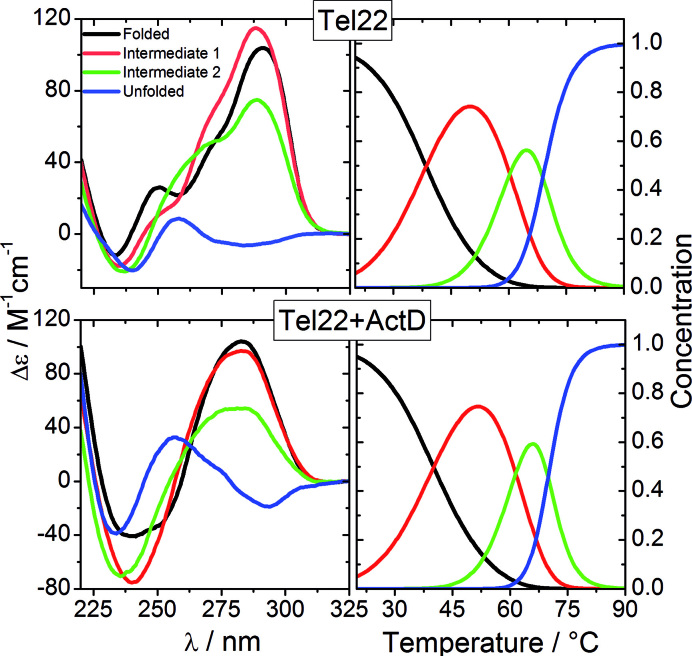
Results of the SVD analysis on CD data of Figure 1. Left panels: spectra of significant species for Tel22 (upper) and Tel22+ActD (lower). Right panels: Relative concentration of significant species as function of temperature for Tel22 (upper) and Tel22+ActD (lower).

We deployed a multi-technique experimental investigation to determine the peculiar structural and molecular properties of Tel22 along a path toward the thermal unfolding, and to compare them with those of the same G-quadruplex when it is bound with the model drug ActD. Our SVD analysis on CD data, whose results are reported in Figure 8, brings us to conclude that the melting process of Tel22 is consistent with the presence of two distinct intermediate states I1 and I2, in agreement with previous experimental results (12,48). In K^+^ solution the native state of Tel22 is supposed to be composed of a mix of antiparallel basket-type and hybrid-type distinct conformers (24,49–52). The progressive increase of the shoulder at 260 nm, and the deepening and red-shift of the minimum located at about 230 nm displayed by the spectra of the intermediate states, suggest that there is a change in the populations of the quadruplex arrangements as temperature increases. On the basis of the classification of the CD spectra made by Karsisiotis *et al.* (9), the observed features are congruous with an increasing population of either hybrid conformation (3+1) (group II) or antiparallel one, like the 22mer G_3_(TTAG_3_)_3_T 2KF8 forming only two G-tetrads.

**Figure 9.**
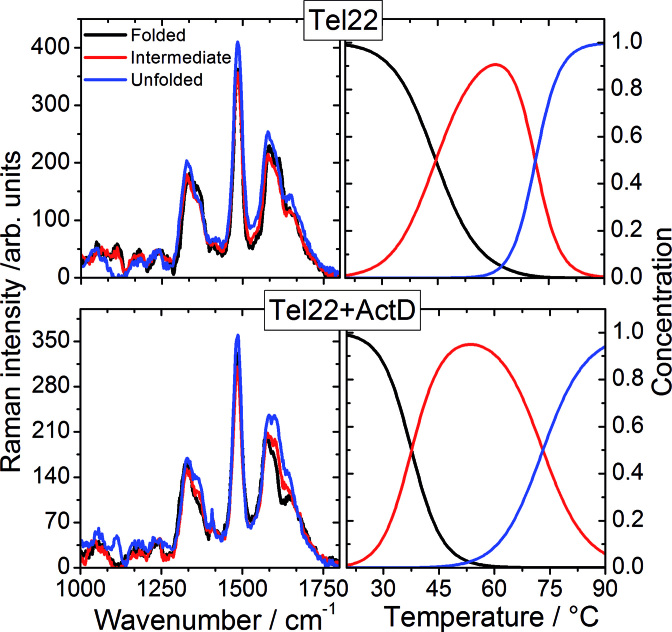
Results of the SVD analysis on UVRR data of Figure 3. Left panels: spectra of significant species for Tel22 (upper) and Tel22+ActD (lower). Right panels: Relative concentration of significant species as function of temperature for Tel22 (upper) and Tel22+ActD (lower). Differently from CD results, SVD analysis identified three significant species during quadruplex thermal unfolding.

The SVD analysis of UVRR data can help discriminating between these two different situations. In particular, we exploit the fact that the gain of intensity of the band at about 1370 cm^−1^, *I*_1370_, which is related to vibrations of T bases (37,41), is correlated with the increasing hydrophobicity of the environment of the thymine C5H_3_ group (53). In Figure 9, where we report the spectra calculated for all the relevant species, it is seen that I_1370_ is smaller in the case of the intermediate state than in the folded one (see also Supplementary Figure S8a where we zoom-in the corresponding spectral region). This hypochromic effect is interpreted as the easier accessibility of the solvent molecules to dT methyl group, which is more promoted in the double-chain reversal than in the lateral/diagonal loops (24). Consequently, our results are consistent with a shift of the conformational equilibrium toward the hybrid (3+1) quadruplex arrangement.

As the temperature further increases, the observed drop of the CD intensity for the I2 state witnesses the progressive separation of G-tetrads, due to the ongoing thermal destabilization of G-quadruplexes, which is also in agreement with the increasing radius of gyration (see Figure 7). At the highest temperatures, a completely unfolded state is attained, characterized by the absence of any stacking between the bases of the oligonucleotide and a spatial arrangement consistent with that of a nucleotide chain obeying the self-avoiding walk statistics (47). Both the values of *R*_g_ for the native and the unfolded states are in agreement with the estimate provided by the mesoscopic model of G-quadruplex thermal stability (54).

Concerning the complex native state, the shift of the conformational equilibrium toward a parallel arrangement emerging from CD data is further supported by the drop of the intensity of the band at 1370 cm^−1^ after complexation, resulting in the decreased hydrophobicity of C5H_3_ thymine group (Supplementary Figure S8b). We hypothesize that the parallel conformation is preferred to the antiparallel or hybrid ones because the former do not have edgewise or diagonal loops possibly hindering end-stacking of ActD to the extremal quadruplex G-tetrad. It is worth of notice that the all-parallel orientation of the phosphate backbones and an opening up of the tetrads on the 3′ and 5′ surfaces is in keeping with the ligand-induced G-quadruplex dimerization observed by the present SANS measurements, as lateral/diagonal loops of hybrid and antiparallel folds could hamper dimer formation. Recently it has been suggested that binding of ligands with low quadruplex selectivity over duplexes does not induce significant conformational changes (55). However, despite its lack of specificity for Tel22 G-quadruplex, ActD has a high enough affinity to promote Tel22 conformational switching, as testified by the topological rearrangement after complexation. In fact ActD (see Supplementary Figure S1), apart from the π-stacking interaction between its phenoxazone ring and the extremal G-quartet surface, can make several hydrogen bonds between its pentapeptide side chains and the quadruplex grooves. This interaction with the phosphodiester backbones defining the grooves stabilizes a different topology with respect to the initial one. This stabilization is also fostered by the ligand conformational flexibility, whose cyclic pentapeptide arms can easily change their arrangement in order to fit into the quadruplex grooves without breaking essential Tel22+ActD hydrogen bonds, as it happens for dsDNA+ActD complexes (17). Within this context, also ActD-driven dimerization of Tel22 has been proposed to be related to the ligand conformational degrees of freedom, with the phenoxazone ring sandwiched between the two quadruplex units and each one of the pentapeptide arms recruiting either the top or the bottom quadruplex (20).

Additional conformational changes take place in the complex with increasing temperature, as shown by the slight rise of *I*_1370_ on passing from the native to the intermediate state (see Supplementary Figure S8c). A partial loss of the parallel arrangement population can be at the origin of this trend, which in turn could be related to the dimer-to-monomer transition revealed by SAXS data.

**Figure 10.**
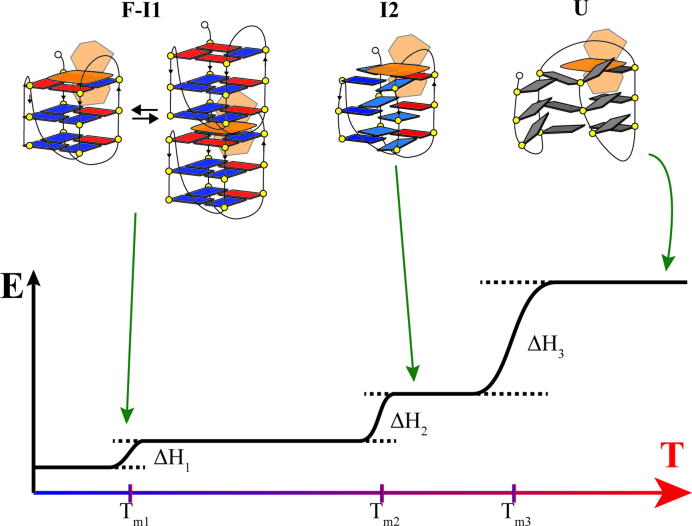
Unfolding picture for Tel22+ActD. Here a possible scenario describing the thermal melting pathway is shown. The Folded and Intermediate 1 states present a high level of order, with stacked G-tetrads (colored in red and blue). Actinomycin D (colored in orange) is end-stacking over the extremal tetrads. After the last transition (T_m3_ as shown by SVD in CD data) the correlation between tetrads is lost, but the interaction of nucleobases with the drug allows the persistence of a compact, although disordered, structure.

With regard to the complex high-temperature structure, the melting of G-tetrads is confirmed not only by the striking change of the CD spectra, but also by the downshift trend of the wavenumber of Raman bands B and C, which are markers for G-quadruplex formation (41). Here, however, the most interesting point is that the average size of the Tel22+ActD complex does not change significantly over the whole temperature range, despite remarkable structural rearrangements do take place. Supplementary Figure S9 shows that a compact fold is retained for the Tel22+ActD complex even after the melting, as illustrated by the 3D models based on *ab-initio* calculations from SAXS data. Our multitechnique approach leads to the picture where the Tel22+ActD complex forms a compact unfolded state, with a significant degree of residual base stacking, but without any remaining G-tetrad element. Quite interestingly, it has been recently shown that even after chemical denaturation by urea a high degree of residually stacked nucleobases is present in single stranded DNA and RNA (56) and G-quadruplex (57). The peculiar compact high-temperature state of Tel22+ActD seems to be somehow stabilized by the persistent stacking of the ActD phenoxazone ring with guanine basis, and by the formation of several hydrogen bonds between the two ActD penta-peptide arms with the bases belonging to guanine runs or to loops. As represented in Figure 10, the emerging view is that the interaction between ActD and Tel22 is not only able to perturb the equilibrium between coexisting conformations in the temperature range where the quadruplex is still in its folded state, but can also preserve a compact structure in conditions where Tel22 undergoes thermal unfolding. These findings prove that ActD is able to set conformational constraints to the complex even in the presence of large structural fluctuations. The persisting binding between ActD and the unfolded Tel22 is an evidence of the capability of this ligand to profit from its remarkable conformational flexibility to optimize the interaction with ssDNA. The ability of ActD to form non-quadruplex compact structures even in single stranded G-rich 3′ DNA sequences may be of importance for the transcriptional regulation within promoter regions of oncogenes.

## CONCLUSIONS

We presented and discussed the results of an experimental study on the structural, molecular and thermodynamic properties of the G-quadruplex formed by the human telomeric sequence AG_3_(TTAG_3_)_3_ in the presence of the ligand ActD, upon thermal unfolding. Singular value decomposition analysis applied to circular dichroism and UV resonance Raman scattering spectra allowed us to identify a temperature region populated with intermediate conformers along the path from the native to the unfolded state, for both the quadruplex alone and the complex. The intermediate states have an increasing hybrid (3+1) character in the former, while the latter has a prevalent parallel nature. We found evidence for a complex unfolded state with persistent residual stacking. The complexed state shows less intense guanine-related bands with respect to the free state, this hypochromic effect supporting the view of the drug binding mechanism as an end-stacking upon the terminal G-tetrad of Tel22. In general, the interaction with the drug seems to make more effective the stacking of guanine basis over the whole investigated temperature range. SANS is decisively used to provide evidence for ligand-induced quadruplex dimerization. On the other hand, SAXS is exploited to characterize the dimer dissociation and the trend of the characteristic size of Tel22 and Tel22+ActD with the increasing temperature. It is seen that, as thermal unfolding proceeds, the Tel22 structure approaches the one of an oligonucleotide with self-avoiding random-coil conformation. On the contrary, the Tel22 complexed with ActD shows a quite compact structure even in its unfolded state. The results from the present integrated experimental strategy suggest that, even at high temperatures, the complex is prevented to adopt a self-avoiding random-coil conformation due to the interaction with the drug, that possibly stabilizes a structure where the oligonucleotide is partially folded. We propose that the ligand conformational flexibility plays a key role in promoting quadruplex topology changes and dimerization, and in stabilizing high-temperature structure with residual base stacking.

## Supplementary Material

Supplementary Data
