# Supplementary material for: Structure of human telomere G-quadruplex in the presence of a model drug along the thermal unfolding pathway
Source: Nucleic Acids Res. 2018 Nov 8;46(22):11927–38. doi: 10.1093/nar/gky1092 (PMC6294516; doi:10.1093/nar/gky1092)
Supplement: Supplementary Data [file gky1092_supplemental_files.pdf]

Structure of human telomere G-quadruplex in the presence of a model drug along the thermal unfolding pathway

F. Bianchi, L. Comez*, R. Biehl, F. D'Amico, A. Gessini, M. Longo, C. Masciovecchio, C. Petrillo, A. Radulescu, B. Rossi, F. Sacchetti, F. Sebastiani, N. Violini and A. Paciaroni**

*comez@iom.cnr.it, **alessandro.paciaroni@unipg.it

Supplementary information

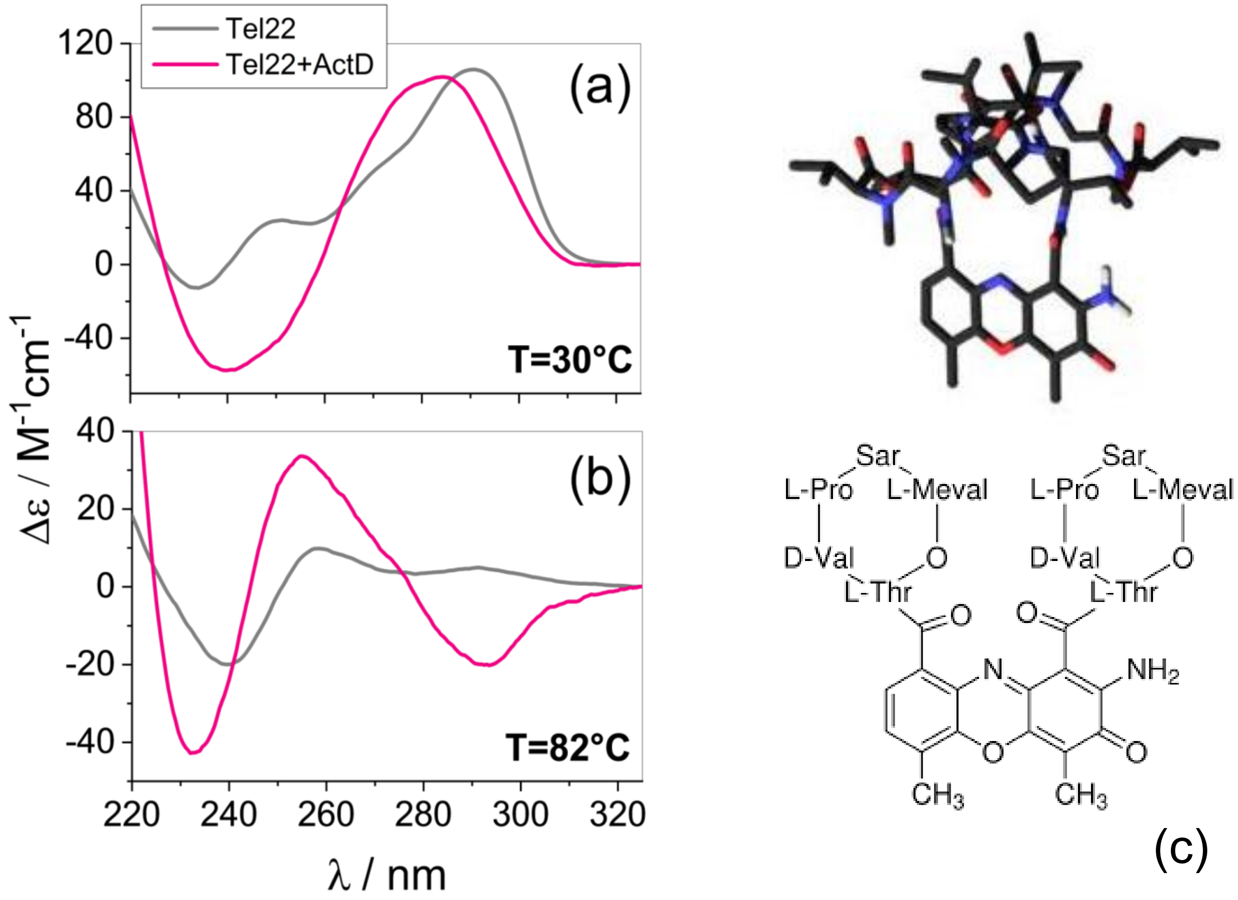

Figure S1: Comparison between CD spectra of Tel22 and Tel22+ActD in the folded (a) and unfolded (b) case, selected from Fig.1. The CD signal of Actinomycin D (ActD) has been subtracted. (c) Schematic molecular structure and formula of ActD.

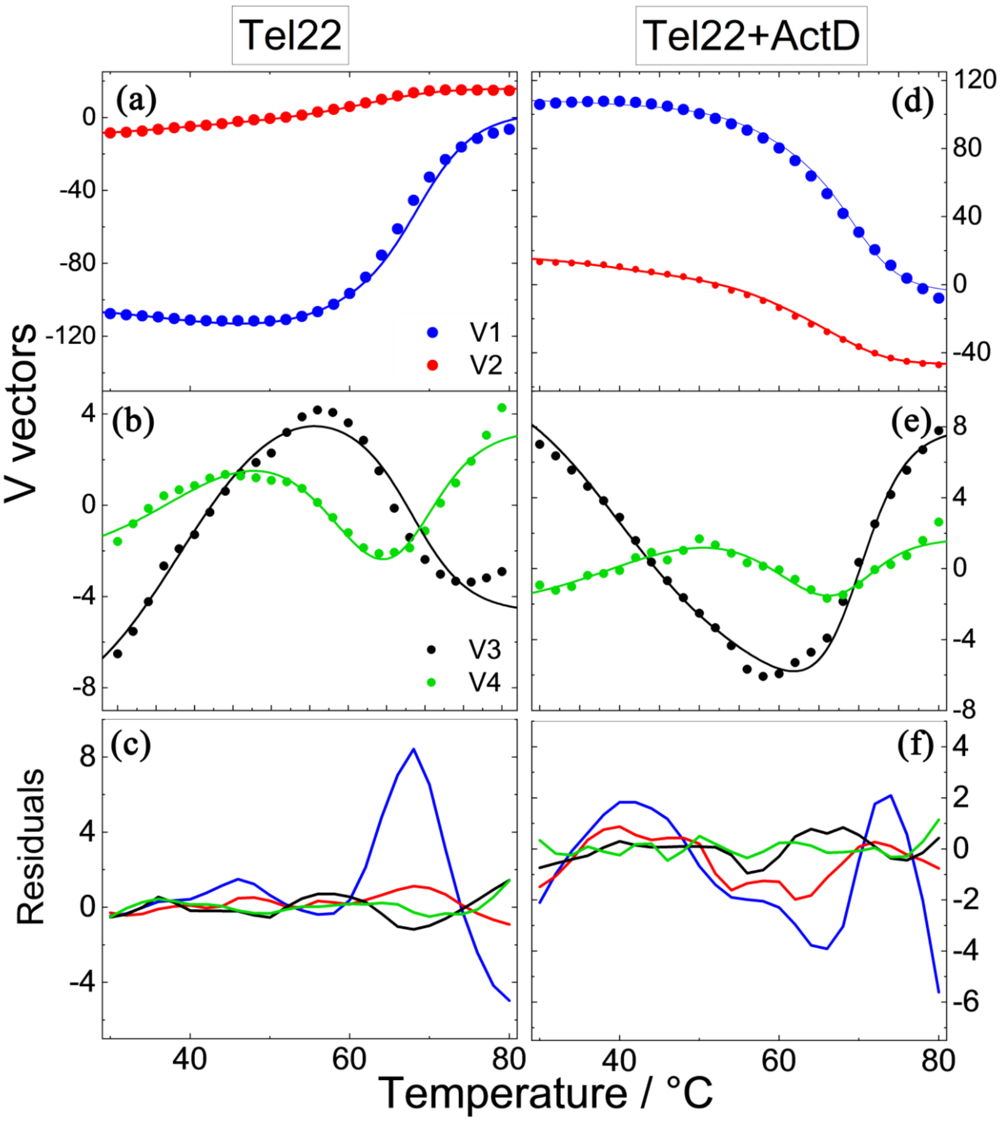

Figure S2: SVD analysis of CD data set in Fig. 1, for Tel22 and Tel22+ActD. Panel (a) and (b): Vectors V1-V4 (full dots) as function of temperature for Tel22. The lines represent the global non-linear fit of the data set to the function introduced by Gray et al. (R1, supporting information), concerning sequential transitions between four spectral species: N ↔ I1 ↔ I2 ↔ U. Panel (c): plot of the residuals of the fit for the four vectors. Panels (d), (e) and (f): same analysis as in (a), (b) and (c) for Tel22+ActD.

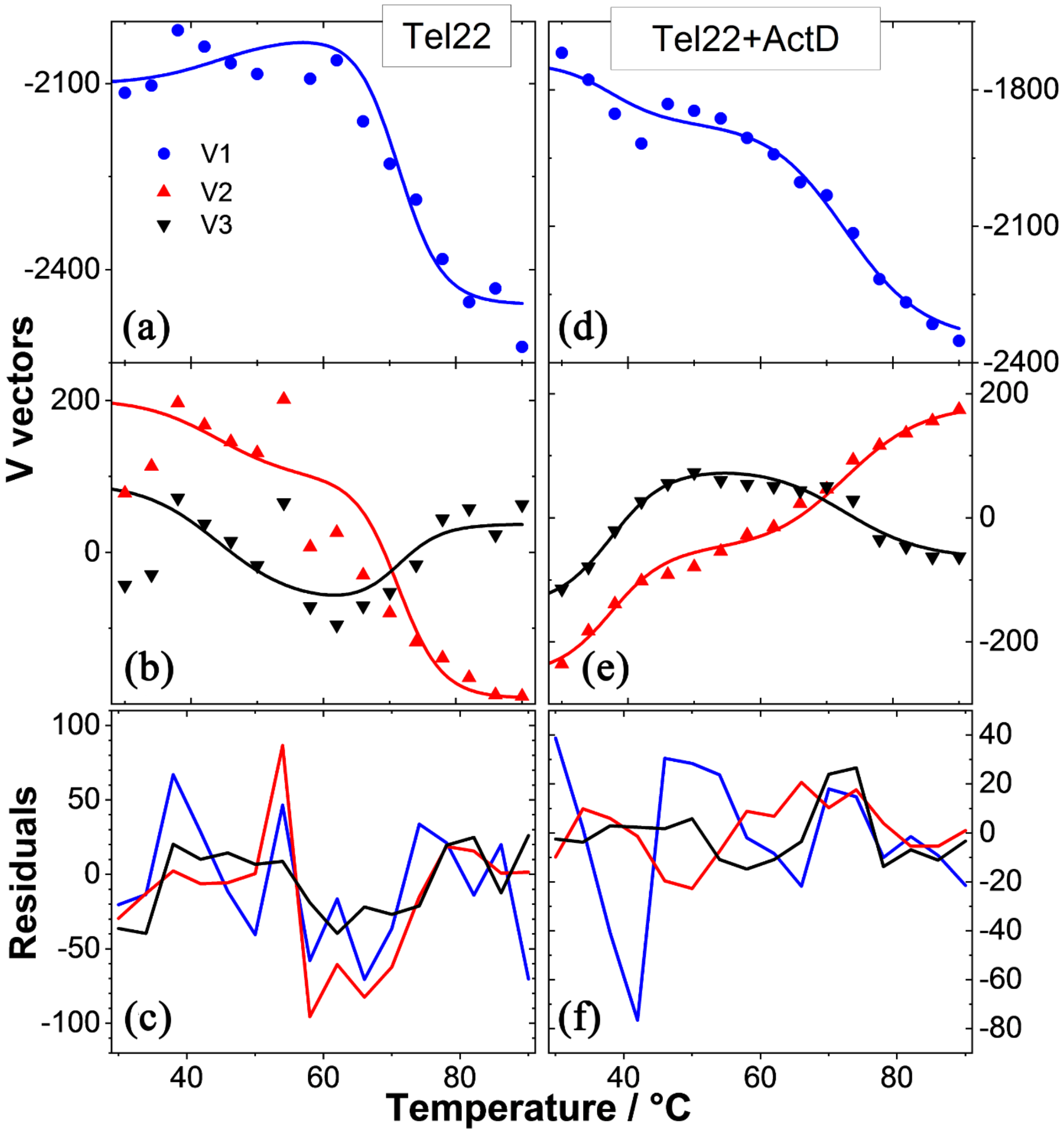

Figure S3: SVD analysis of UVRR data set in Fig. 3, for Tel22 and Tel22+ActD. Panel (a) and (b): Vectors V1-V3 (full dots) as function of temperature for Tel22. The lines represent the global non-linear fit of the data set to a modified version of the function introduced by Gray et al. (R1), concerning sequential transitions between three spectral species: N ↔ I1 ↔ U. Panel (c): Plot of the residuals of the fit for the three vectors. Panels (d), (e) and (f): same analysis as in (a), (b) and (c) for Tel22+ActD.

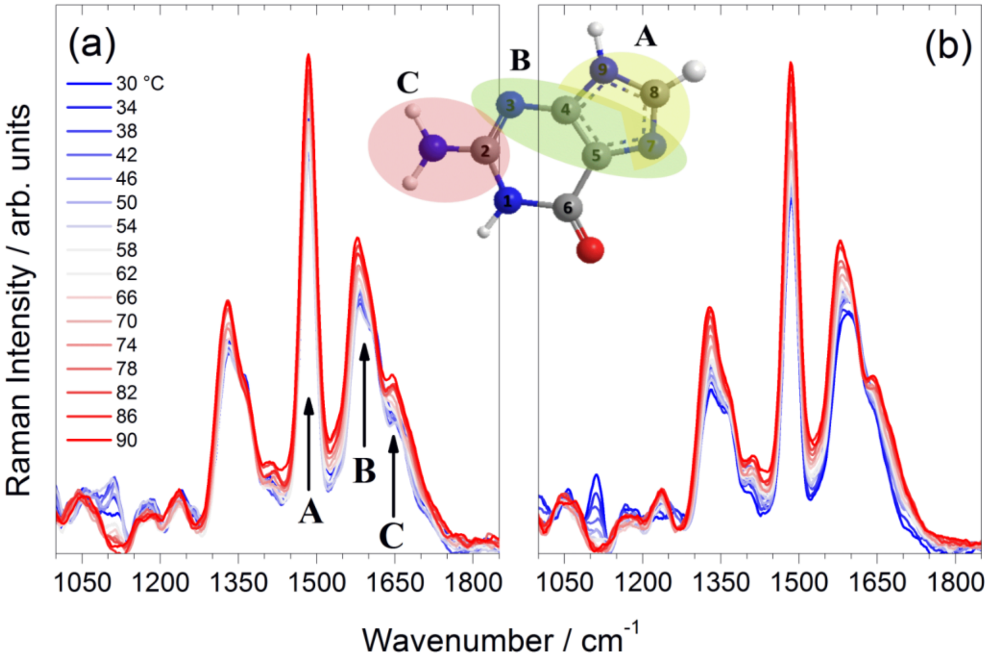

Figure S4: Temperature dependent UVRR spectra, excited at λ_e=250 nm, collected for (a) Tel22 45 μM in 150 mM KCl solution and (b) Tel22+ActD 1:2, 45 μM in 150 mM KCl solution. The arrows highlight the three bands mainly assigned to normal in-plane modes of dG residues, as represented in the central picture.

SVD Tables

Table S1: Results for the SVD calculation on CD data of Fig. 1 for Tel22 (G-quadruplex) and Tel22+ActD (complex), for the first five V vectors. The values indicate that only four components should be taken into account, since the first four singular values contribute to 98.56% and 98.04% of the total variance for Tel22 and Tel22+ActD, respectively.

Index	Singular value		% of total variance		V matrix autocorrelation coefficient	
	G-quadruplex	Complex	G-quadruplex	Complex	G-quadruplex	Complex
1	512.04	473.07	85.45	72.72	0.9764	0.9737
2	51.98	133.60	8.67	20.54	0.9405	0.9298
3	18.46	25.54	3.08	3.93	0.8899	0.8988
4	8.17	5.50	1.36	0.85	0.8214	0.8349
5	1.59	1.98	0.27	0.30	0.5676	0.3386

Table S2: Results for the SVD calculation on UVRR data of Fig. 3 for Tel22 (G-quadruplex) and Tel22+ActD (complex), for the first five V vectors.

Index	Singular value		% of total variance		V matrix autocorrelation coefficient	
	G-quadruplex	Complex	G-quadruplex	Complex	G-quadruplex	Complex
1	8784.3	8026.0	79.33	78.79	0.9293	0.9337
2	552.4	485.3	4.99	4.76	0.9079	0.7884
3	214.9	233.8	1.94	2.30	0.6970	0.7228
4	147.9	139.5	1.34	1.37	-0.3096	-0.1543
5	138.3	131.8	1.25	1.29	-0.2713	-0.3921

FORM FACTORS AND DIMERIZATION

The SANS coherent macroscopic scattering cross section Σ_c (scattering intensity in an absolute scale) of a simple scattering system consisting of a diluted solution of particles can be described as (R2) :

$$\frac{d\Sigma_c(Q)}{d\Omega} = (\frac{N}{V})V_p^2 \Delta\rho^2 P(Q) \quad \text{(S1)}$$

where (N/V) is the number density of particles, V_P is the particle volume, $\Delta\rho^2$ is the contrast factor, and P(Q) is the single particle form factor, with the conditions P(Q→0) = 1, P(Q→∞) = 0. P(Q) depends on the size, shape and internal structure of the particle and can be calculated analytically of numerically for elementary shapes (R2). To characterize the large-scale properties of Tel22 and Tel22+ActD, their SANS intensities have been normalized to 1 in the range of the smallest Q values (See Fig. 5). In the case of Tel22, Eq. (S1) reads as:

$$(\frac{d\Sigma_c(Q)}{d\Omega})_{Tel22} = (\frac{N_{Tel22}}{V})V_{Tel22}^2 \Delta\rho_{Tel22}^2 P_{Tel22}(Q) \quad \text{(S2)}$$

where N_{Tel22} is the number of free G-quadruplex in solution, and $\Delta\rho_{Tel22}^2$ and $P_{Tel22}(Q)$ are the contrast factor and the form factor for Tel22. By using the SASVIEW software (R3) the form factor of a squared parallelepiped with side 15±1 Å and height 27±1 Å has been seen to provide an excellent fit to the trend of the SANS intensity from Tel22 (See Fig. 5,). To describe the SANS intensity from Tel22+ActD we used a form factor consisting of a mixture of monomers (squared parallelepiped) and dimers (two adjacent squared parallelepipeds), representing Tel22+ActD complexes with molecularity 1:1 and 2:1 respectively. It is easy to see that, under the approximation that the dimer molecular volume is about twice the monomer molecular volume, i.e. the ligand molecular volume is neglected, the coherent SANS cross section of the complex in dilute conditions is given by the equation:

$$(\frac{d\Sigma_c(Q)}{d\Omega})_{Tel22+ActD} = (\frac{N_{Tel22}}{V})V_{Tel22}^2 \Delta\rho_{Tel22}^2[(1-f)P_{Mono}(Q) + 2fP_{Dimer}(Q)] \quad \text{(S3)}$$

where *f* is the fraction of monomers, i.e. Tel22 units, undergoing dimerization. Eq. (S3) describes in an excellent way the SANS data (see Fig. 5), with monomer characteristic sizes that are very similar to those of unbound Tel22 (15±2 Å and height 28±2 Å). In the limit of small Q, Eq. (S2) and (S3) become:

$$(\frac{d\Sigma_c(Q)}{d\Omega})_{Tel22} = (\frac{N_{Tel22}}{V})V_{Tel22}^2 \Delta\rho_{Tel22}^2 \quad \text{(S4)}$$

$$(\frac{d\Sigma_c(Q)}{d\Omega})_{Tel22+ActD} = (\frac{N_{Tel22}}{V})V_{Tel22}^2 \Delta\rho_{Tel22}^2(1+f) \quad \text{(S5)}$$

Eq. (S4) and (S5) provide very good estimates for the measured absolute intensity as Q→0, as shown in Fig. S5.

POROD INVARIANT

The Porod invariant I_P is a model-independent quantity that can be calculated from the intensity dΣ/dΩ of either SANS or SAXS experiments through the relationship (R4):

$$I_P = \frac{\int d\Sigma(Q)}{d\Omega} Q^2 dQ \quad \text{(S6)}$$

Provided that background is very accurately subtracted, from I_P one can estimate the particle molecular volume V_p:

$$V_p = 2\pi \frac{d\Sigma(0)}{d\Omega} / I_P \quad \text{(S7)}$$

KRATKY PLOT

The Kratky plot is the graphical representation of the curve Q^2(dΣ(Q)/dΩ) as a function of Q. It is employed to qualitatively distinguish folded and unfolded states of biomolecules (R5). The scattering intensity from a folded biomolecule decays at high angles approximately as ~ Q^{-4}, which implies a bell shape with a well-defined maximum for the Kratky plot. Conversely, the trend of unfolded biomolecules is associated to that of an ideal Gaussian chain, whose scattering intensity has a ~ Q^{-2} asymptotic behaviour, with the consequent plateau at large Q values for the

relevant Kratky plot. In Fig. S7 it is represented the Kratky plot from SAXS data for Tel22 and Tel22+ActD as a function of temperature.

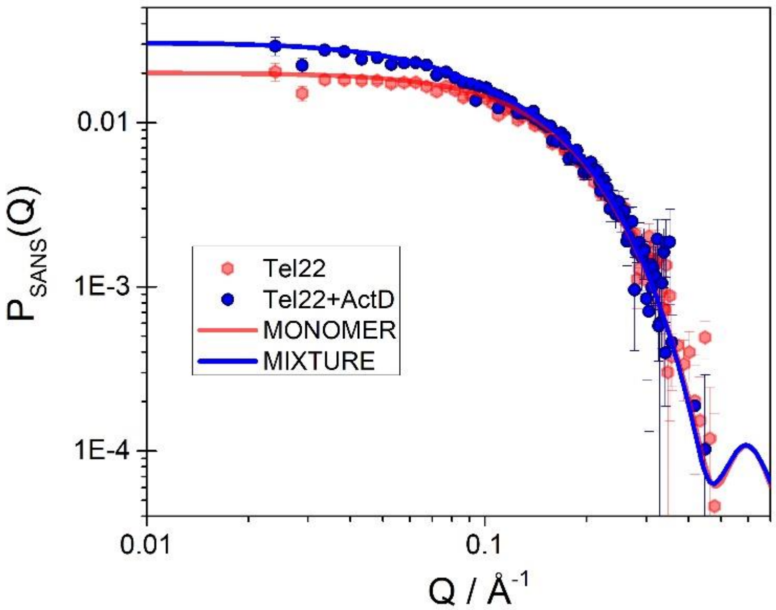

Figure 5: Absolute SANS intensities for Tel22 (closed red hexagones) and Tel22+ActD (closed blue circles) form factors, P(Q). The form factors of a parallelepiped representing the Tel22 monomer (red line) and a mixture of parallelepiped monomers and dimers fitted to the complex data (blue line) are also reported. Details on the calculation for monomer and dimer form factors are given in the text.

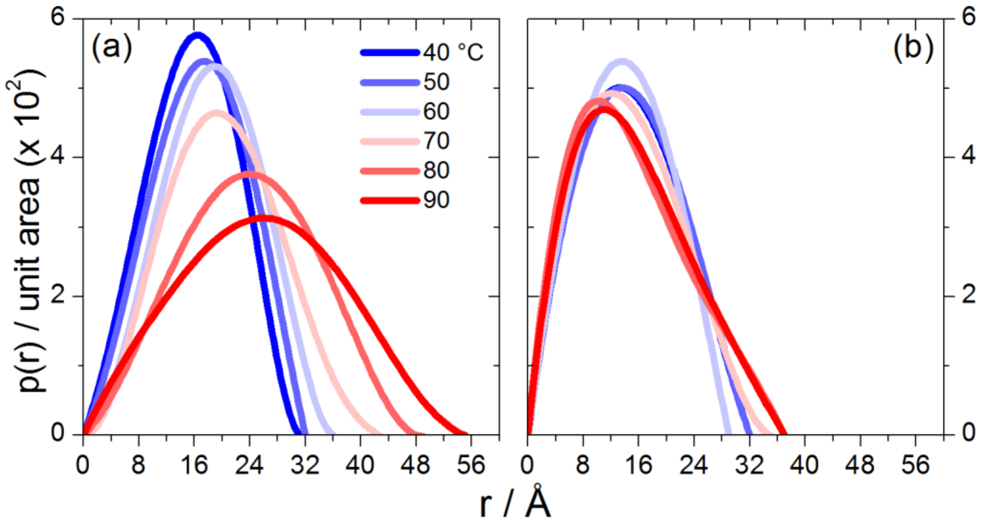

Figure S6: Pair distance distribution function calculated by GNOM. Panel (a): p(r) for Tel22. Panel (b): p(r) for Tel22+ActD.

NOTE: No direct information on the molecularity of the complex can be obtained from the shape of the SAXS form factor, due to the lower concentration of the samples and the shorter duration of SAXS measurements compared to the SANS experiment.

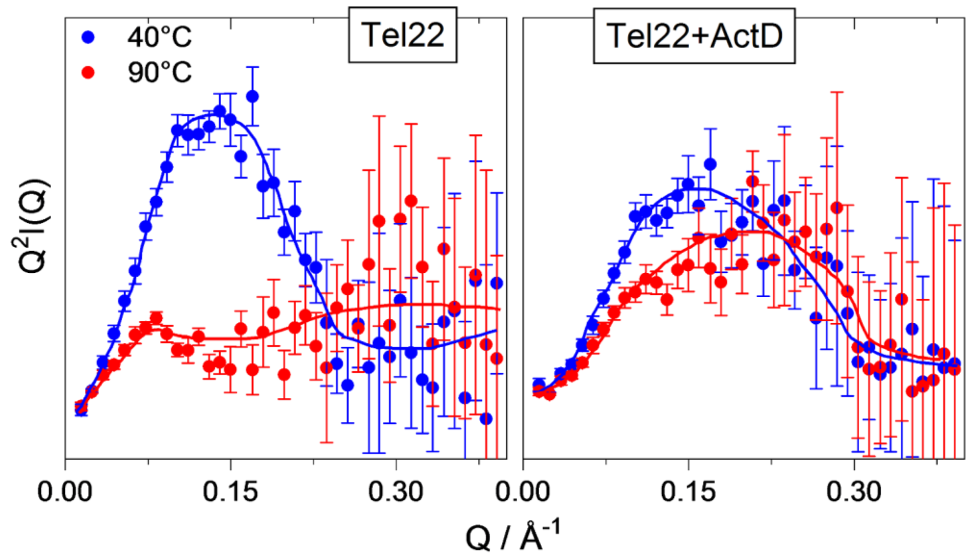

Figure S7: Kratky plot of SAXS data for Tel22 and Tel22+ActD. Lines are only a guide for the eye.

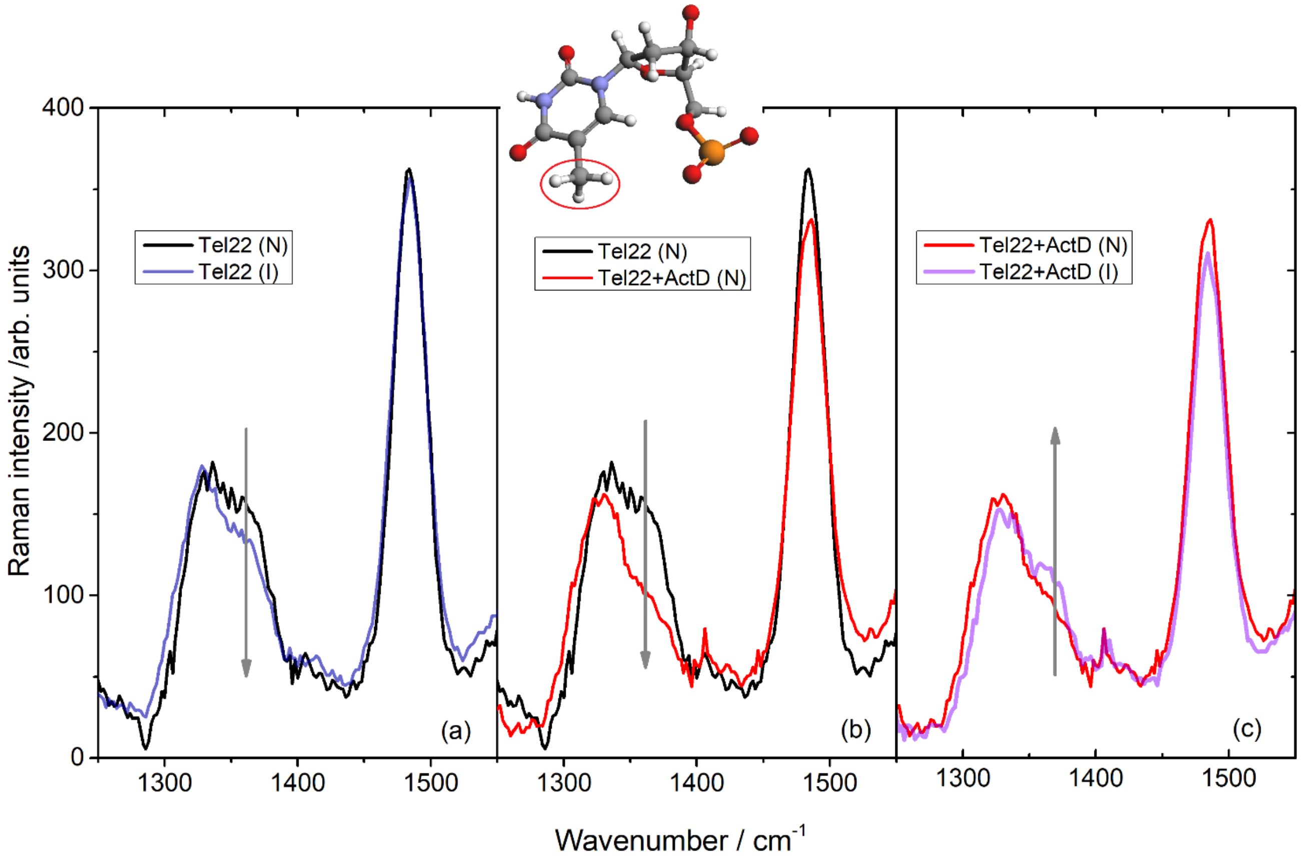

Figure S8: SVD UVRR spectra of Tel22 and Tel22+ActD: the intensity decrease of the band at 1370 cm^{-1} is interpreted as a major accessibility of the solvent molecules to dT methyl group (highlighted in the cartoon). Comparison of Tel22 in the folded and intermediate states (a), of Tel22 and Tel22+ActD in the native state (b), and of Tel22+ActD in the folded and intermediate states (c).

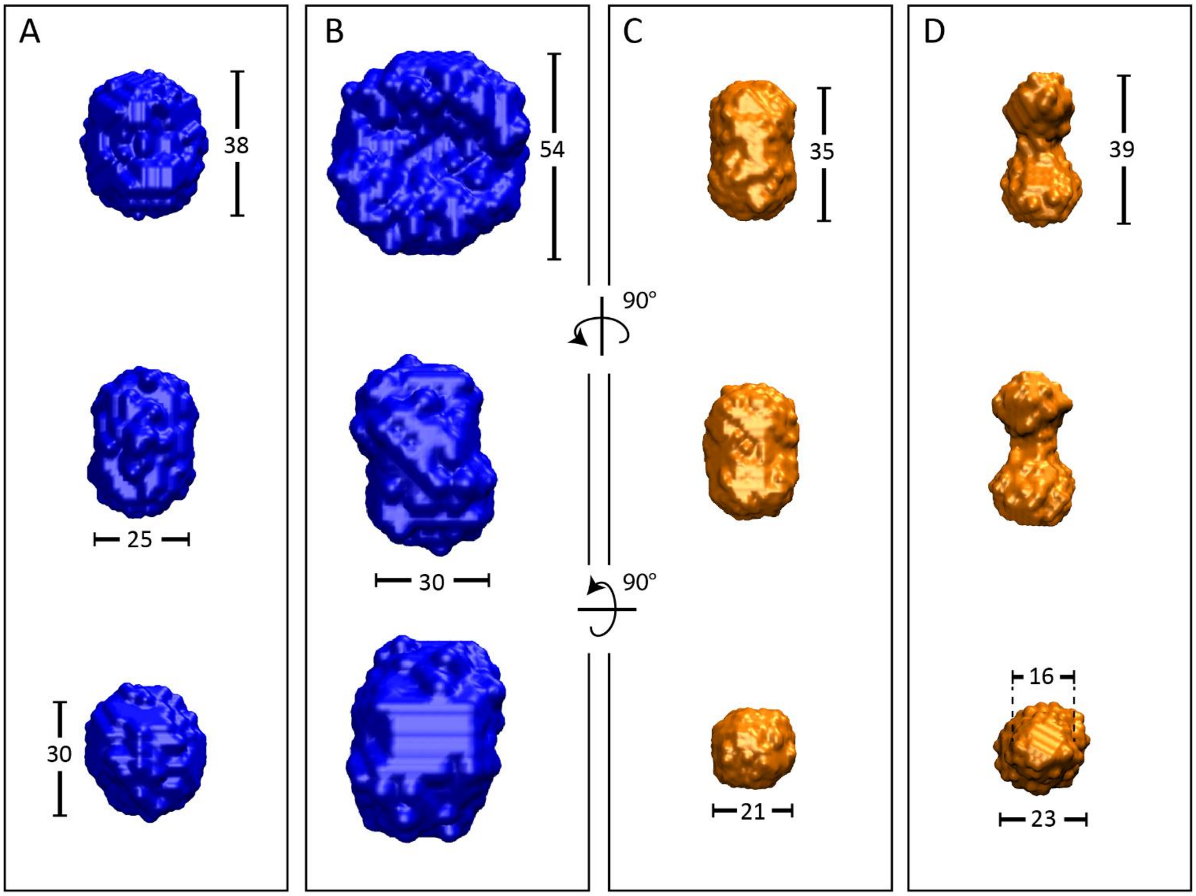

Figure S9: Dummy atom models obtained by the ATSAS suite with ab-initio calculations (see Materials and Methods). Panels A, B, C and D show Tel22 at 40 °C, Tel22 at 90 °C, Tel22+ActD at 40 °C and Tel22+ActD at 90 °C, respectively. Measures are expressed in Angstrom. Arrows indicate the direction of rotation from upper to lower models. The enlargement of Tel22 sample upon melting is clear, as the persistence of a compact structure for Tel22+ActD, even after melting.

References

(R1) Gray, R.D., Buscaglia, R. and Chaires, J.B. (2012) Populated Intermediates in the Thermal Unfolding of the Human Telomeric Quadruplex. *J. Am. Chem. Soc.*, 134 (40), 16834.

(R2) Hammouda, B. *SANS Toolbox*: http://www.ncnr.nist.gov/staff/hammouda/the_SANS_toolbox.pdf

(R3) http://www.sasview.org/

(R4) Small angle x-ray scattering. Von O. GLATTER und O. KRATKY. London: Academic Press Inc. Ltd. 1982, ISBN 0-12-286280-5.

(R5) Kikhney, A. G. and Svergun, D. I. (2015) A practical guide to small angle X-ray scattering (SAXS) of flexible and intrinsically disordered proteins. *FEBS Lett.*, 589, 2570-2577.
